# Decreased expression of mitochondrial aminoacyl-tRNA synthetases causes downregulation of OXPHOS subunits in type 2 diabetic muscle

**DOI:** 10.1016/j.redox.2023.102630

**Published:** 2023-02-08

**Authors:** Iliana López-Soldado, Adrian Gabriel Torres, Raúl Ventura, Inma Martínez-Ruiz, Angels Díaz-Ramos, Evarist Planet, Diane Cooper, Agnieszka Pazderska, Krzysztof Wanic, Declan O'Hanlon, Donal J. O'Gorman, Teresa Carbonell, Lluís Ribas de Pouplana, John J. Nolan, Antonio Zorzano, María Isabel Hernández-Alvarez

**Affiliations:** a Department de Bioquímica i Biomedicina Molecular, Facultat de Biología, 08028, Spain; b Institut de Biomedicina de la Universitat de Barcelona IBUB, Barcelona, Spain; c Institute for Research in Biomedicine (IRB Barcelona), the Barcelona Institute of Science and Technology, Barcelona, Spain; d CIBER de Diabetes y Enfermedades Metabólicas Asociadas (CIBERDEM), Instituto de Salud Carlos III, Spain; e Metabolic Research Unit, St James's Hospital, and Trinity College, Dublin, Ireland; f National Institute for Cellular Biotechnology, 3U Diabetes Partnership & School of Health and Human Performance, Dublin City University, Dublin, Ireland; g Departament de Biologia Cel·lular, Fisiologia i Immunologia, Facultat de Biologia, 08028, Barcelona, Spain

**Keywords:** Type 2 diabetes, Mitochondrial aminoacyl tRNA synthetases, Nitrosative stress, OXPHOS, Skeletal muscle, Nitric oxide

## Abstract

Type 2 diabetes mellitus (T2D) affects millions of people worldwide and is one of the leading causes of morbidity and mortality. The skeletal muscle (SKM) is one of the most important tissues involved in maintaining glucose homeostasis and substrate oxidation, and it undergoes insulin resistance in T2D. In this study, we identify the existence of alterations in the expression of mitochondrial aminoacyl-tRNA synthetases (mt-aaRSs) in skeletal muscle from two different forms of T2D: early-onset type 2 diabetes (YT2) (onset of the disease before 30 years of age) and the classical form of the disease (OT2). GSEA analysis from microarray studies revealed the repression of mitochondrial mt-aaRSs independently of age, which was validated by real-time PCR assays. In agreement with this, a reduced expression of several encoding mt-aaRSs was also detected in skeletal muscle from diabetic (db/db) mice but not in obese ob/ob mice. In addition, the expression of the mt-aaRSs proteins most relevant in the synthesis of mitochondrial proteins, threonyl-tRNA, and leucyl-tRNA synthetases (TARS2 and LARS2) were also repressed in muscle from db/db mice. It is likely that these alterations participate in the reduced expression of proteins synthesized in the mitochondria detected in db/db mice. We also document an increased iNOS abundance in mitochondrial-enriched muscle fractions from diabetic mice that may inhibit aminoacylation of TARS2 and LARS2 by nitrosative stress.

Our results indicate a reduced expression of mt-aaRSs in skeletal muscle from T2D patients, which may participate in the reduced expression of proteins synthesized in mitochondria. An enhanced mitochondrial iNOS could play a regulatory role in diabetes.

## Highlights

• ·Mt-aaRSs are downregulated in the skeletal muscle of type 2 diabetic patients and diabetic mice, affecting the synthesis of ND2 which is a mitochondrially encoded subunit of complex I. .

## Graphical abstract

**Figure:**
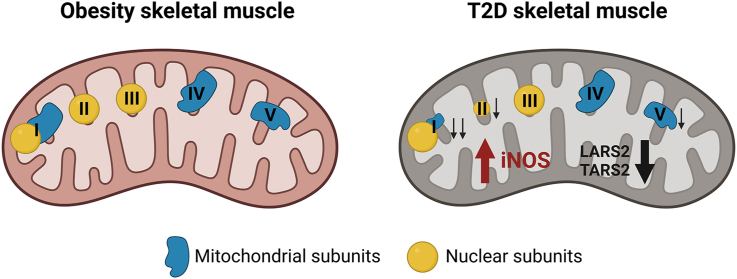

## Abbreviations

ATP5A: ATP synthase F1 subunit Alpha
BCAAs: branched chain aminoacids
CARS2: cysteynil-tRNA synthetase 2
DARS2: aspartyl-tRNA synthetase 2
eNOS: endothelial nitric oxide synthase
GARS: glycine-tRNA synthetase
GSEA: gene Set Enrichment Analysis
HARS2: histidyl-tRNA synthetase 2
IARS2: isoleucyl-tRNA synthetase 2
iNOS: inducible nitric oxide synthase
KARS: lycine-tRNA synthetase
LARS2: leucyl-tRNA synthetase 2
MARS2: methionyl-tRNA synthetase 2
MT-ND2: NADH-dehydrogenase subunit 2
mt-aaRSs: mitochondrial aminoacyl-tRNA synthetases
MT-CO1: mitochondrial cytochrome *c* oxidase subunit 1
MT-ATP6: mitochondrial ATP synthase F0 subunit 6
MT-ND2: mitocondrial NADH:Ubiquinone Oxidoreductase Core Subunit 2
NARS2: asparaginyl-tRNA synthetase 2
NDUFB8: NADH:Ubiquinone Oxidoreductase Subunit B8
NO: nitric oxide
PARS2: prolyl-tRNA synthetase 2
RNS: reactive nitrogen species
VARS2: valyl-tRNA synthetase 2
OT2: classical type 2 diabetes
OXPHOS: oxidative phosphorylation system
PCA: principal component analysis
ROS: reactive oxygen species
SARS2: serine-tRNA synthetase 2
SDHB: succinate dehydrogenase iron-sulfur subunit
TARS2: threonyl-tRNA synthetase 2
T2DM: type 2 diabetes mellitus
UQCRC2: Ubiquinol-Cytochrome C Reductase Core Protein 2
YT2: early-onset type 2 diabetes
YARS2: tyrosyl-tRNA synthetase 2
WARS2: tryptophanyl-tRNA synthetase 2

## 1 Introduction

The prevalence of type 2 diabetes (T2D) has reached epidemic proportions worldwide. Furthermore, T2D is related to many chronic comorbidities that can undermine the quality of life and life span [1]. T2D is characterized by insulin resistance (IR) in skeletal muscle [2], which can result from combining a genetic predisposition with obesity, a sedentary lifestyle, and an unhealthy diet [3].

Mitochondria are the major functional components of cellular fuel oxidation and ATP production [4]. The preliminary evidence linking mitochondrial dysfunction to insulin resistance in skeletal muscle comes from studies performed in non-insulin-dependent diabetic individuals who exhibited an increased glycolytic to oxidative ratio, which suggests that a dysregulation between mitochondrial oxidative capacity and capacity for glycolysis is an important component of the expression of insulin resistance [5]. Later on, it was described that the expression of many genes of oxidative metabolism is reduced in T2D [6,7]. Among them, PPARγ coactivator 1-α and -β (PGC1-α and PGC1-β) is decreased in both diabetic subjects and family history-positive nondiabetic subjects [6]. Also, the total activity of mitochondrial electron transport in the skeletal muscle is reduced in T2DM subjects [8]. Moreover, the skeletal muscle mitochondrial proteins are downregulated in the transition from prediabetes into type 2 diabetes [9]. Additionally, insulin resistance in the skeletal muscle of insulin-resistant offspring of patients with type 2 diabetes is associated with dysregulation of intramyocellular fatty acid metabolism, possibly because of an inherited defect in mitochondrial oxidative phosphorylation [10]. Despite the well-documented association between mitochondrial dysfunction and insulin resistance, whether impaired mitochondrial oxidative capacity is causal to or is a consequence of insulin resistance remains a matter of debate [11].

Mitochondrial protein synthesis provides key components of the oxidative phosphorylation complexes. For mitochondrial protein synthesis, a collaboration of two genomes is required: mitochondrial tRNAs and ribosomal RNAs are encoded by the mitochondrial genome, whereas all protein factors involved in the translation of mitochondrial proteins are encoded by nuclear genes, translated in the cytoplasm and imported into mitochondria.

The mitochondrial aminoacyl-tRNA synthetase proteins (mt-aaRSs) are a group of nuclear-encoded enzymes that facilitate the conjugation of each of the 20 amino acids to its cognate tRNA molecule, through a two-step reaction, where they first activate the amino acid with ATP, forming an intermediate aminoacyl-adenylate, and then transfer the aminoacyl group to the 3ʾ-end of its own tRNA [12]. These mt-aaRSs are sometimes encoded by the same genes as the cytosolic ones (dualy-localized mt-aaRS), yet in many cases, are encoded by a different gene (exclusively localized) [13]. In humans, only two aaRs are dualy-localized (Glycil- and lysil-tRNA synthetase). Each of the 19 human genes encoding mt-aaRS have been associated with a wide spectrum of human disorders [14], highlighting their importance for human health [15,16]. The main organs affected are the central nervous system, and the musculoskeletal, cardiovascular, and urinary systems [17].

Recently, it has been described that nitrosative stress inhibits aminoacylation of mitochondrial threonyl-tRNA and leucyl-tRNA synthetases (TARS2 and LARS2) by S-nitrosation [18]. These data clearly showed that mt-aaRSs constitute a family of enzymes sensitive to posttranslational S-nitrosation modification mediated by reactive nitrogen species (RNS) [18]. Protein nitrosation is a recently highlighted protein modification. Nitrosation is a general term that indicates nitrogen-associated gaseous modification and includes the ligation of nitric oxide (NO) or nitrogen dioxide on proteins, peptides, and metal ions [19]. NO is synthesized by NO synthase (NOS) during the catalysis of l-arginine into l-citrulline. Three NOS isoforms have been reported: neuronal NOS (nNOS and NOS1), inducible NOS (iNOS and NOS2), and endothelial NOS (eNOS and NOS3) [20]. Although NO is usually beneficial in the basal context, cumulative stress from chronic inflammation or oxidative insult produces a large amount of NO, which induces atypical protein nitrosation [20].

In this study, we wanted to determine if patients or animal models with type 2 diabetes or obesity presented alterations in the muscle that could be associated with mitochondrial dysfunction and insulin resistance and explore whether mitochondrial nitrosative stress could be involved in these alterations.

## 2 Methods

### 2.1 Study subjects

As previously reported [21], early-onset type 2 diabetes was described as the onset of disease before the age of thirty, while classical onset type 2 diabetes refers to the onset of disease in patients over fifty. Participants of the study who presented with comorbidities or a secondary form of diabetes were not included. All subjects had normal or high fasting c-peptide concentrations at diagnosis (≥2.5 ng/ml), were clinically obese (BMI >30), and were weight-stable and negative for glutamic acid decarboxylase (GAD) antibodies. The exclusion criteria included active cancer, a history of ketoacidosis or ketosis, the use of corticosteroids, and pregnancy. Controls who were healthy, obese, had no family history of diabetes and had normal glucose tolerance were recruited from endocrine clinics at St James’ Hospital and through advertisements published by Dublin City University. All patients gave written informed consent, approved by the local Research Clinics Committee. They were sedentary at baseline (as determined by the International Physical Activity Questionnaire- Long Format), alongside a low VO_2_ max. 46% of patients with early-onset, and 39% with classical-onset type 2 diabetes, had a family history of this disease via a first-degree relative.

Prior to study enrolment, the duration of diabetes was 2.1 years for early-onset disease and 4.8 years for classical-onset disease. Study participants presented with stable blood glucose concentrations, with 88% of the early-onset, and 78% of the classical-onset type 2 diabetes group, being treated with oral hypoglycaemic medications. Insulin was administered to 23% of early-onset and 17% of classical-onset type 2 diabetes patients. All subjects underwent screening, during which a physical examination was carried out, and a medical history was taken. Blood, urine, and its components were analysed. Anthropometric data were obtained by measuring the participant's BMI, weight (upon fasting and taken using a calibrated medical scale), waist-to-hip ratio (measured around the abdomen at the narrowest point between the iliac crests and the lowest costal margin above the umbilicus, as well as around the greater trochanters of the femur and gluteal mass). The patient's height was also taken using a stadiometer. This study forms part of a larger study in which the participants were subjected to various lifestyle changes [22,23]. All methods explained above adhered to the relevant regulations and guidance.

Patients who accessed the diabetes or endocrinology services at St James' Hospital in Dublin and who had either obesity or diabetes were invited to participate in the study. The local Research Ethics Committee approved the study protocol and consent was provided by all subjects. The project was funded with a grant to JJN at 10.13039/501100001637Trinity College Dublin from 10.13039/501100001648EFSD/10.13039/501100004191Novo Nordisk Clinical Research Program in Adolescents with Type 2 Diabetes (Cellular Mechanisms of Insulin Resistance in Early Onset Type 2 Diabtetes, grant reference T04001) with the project being approved by the ‘Joint St James’ Hospital and Adelaide and Meath Hospital Ethics Committee, with a reference number of 2008/06/03.

### 2.2 Muscle biopsies

As previously described [21], all patients were in a fasting state before the muscle biopsy was taken. The biopsies were obtained from the vastus lateralis muscle (100 mg) under local anaesthesia. Specifically, an area of skin was anaesthetised with 1% lidocaine, and a small incision of 0.5 cm was made. To obtain the tissue, a Bergstrom biopsy needle was inserted into the muscle, and approximately 100 mg of tissue was extracted using suction. The samples were snap-frozen in liquid nitrogen and stored at a temperature of −80 °C before their RNA was extracted.

### 2.3 Microarray assays

As previously reported [21], the IRB Functional Genomics Core Faculty gave access to their microarray services, which included quality control testing of total RNA using Nanodrop spectrophotometry, and the Agilent Bioanalyser. cDNA libraries were constructed and amplified from 25 ng of total RNA with 17 cycles of amplification and using WTA2 (Sigma-Aldrich). 8 μg of the cDNA was cut into fragments by the enzyme DNAseI and biotinylated by a terminal transferase, which was obtained from the GeneChip Mapping 250K Nsp Assay Kit (Affymetrix). Following the Affymetrix protocol, a hybridization mixture was prepared with each sample being hybridized to a PrimeView Human array (Affymetrix). As detailed in the Fluidics protocol FS450_002, the arrays were washed and stained in Fluidics station 450 the samples were then scanned using the GeneChip scanner 3000 (both Affymetrix), following the recommendations of the manufacturer. The GCOS software permitted the generation of CEL files using DAT files (Affymetrix). The RMA algorithm 29 was used to determine the normalized expression intensities from Affymetrix CEL files, and microarray pre-processing was carried out using the R package (R core team (2014). Results in Supplementary Table 1 as previously reported [21].

### 2.4 Mouse models

All procedures were approved by the Barcelona Science Park's Animal Experimentation Committee and carried out in accordance with the European Community Council Directive and the National Institute of Health guidelines for the care and use of laboratory animals. Male db/db mice and db/+ littermates (n = 8 per group) were purchased from Charles River Laboratories (Sulzfeld, Germany). Male ob/ob mice and C57BL6/J wild-type littermates (n = 8 per group) were purchased from Charles River Laboratories (Sulzfeld, Germany), and were obtained at the age of 5 weeks. Mice were fed a standard chow diet and water ad libitum and were kept in 12-h dark-light periods. Animals were killed in a non-fasted state at the age of 8 weeks. Mice were anaesthetised using isofluorane and sacrificed by cervical dislocation.

### 2.5 RNA extraction and real-time PCR assays

RNA from muscle in humans and mice was extracted by using a protocol combining Trizol reagent (Invitrogen) and RNeasy Mini Kit columns (Qiagen) following the manufacturer's instructions. RNA was reverse-transcribed with the SuperScriptIII reverse transcriptase kit (Invitrogen). Quantitative polymerase chain reaction (PCR) was performed using SYBER green (Applied Biosystems) or Taqman Universal PCR Mastermix (Applied Biosystems) on the QuantStudio 6 Flex. As endogenous control to correct for potential variation in RNA loading and quantification, Human PPIA (cyclophilin A) (#431883E) and mouse PPIA (Mm02342429_g1) from Applied Biosystems were used. mRNA expression was calculated using the ΔCT method. Briefly, the ΔCT was calculated by subtracting the CT for cyclophilin A, from the CT for the gene of interest. The relative expression of the gene of interest is calculated using the expression 2−ΔCT and reported as arbitrary units. The TaqMan probes used for humans were CARS2 (Hs00226049_m1), MARS2 (Hs00536599_s1), SARS2 (Hs00215168_m1), TARS2 (Hs00372819_m1), NARS2 (Hs00372778_m1), DARS2 (Hs00216620_m1), WARS2 (Hs00210571_m1), HARS2 (Hs00247230_m1), PARS2 (Hs00384448_m1), IARS2 (Hs01058371_m1), LARS2 (Hs01118920_m1) and VARS2 (Hs00383681_m1). For mouse: CARS2 (Mm01209058_m1), LARS2 (Mm00467701_m1), PARS2 (Mm01954197_s1), NARS2 (Mm00841042_m1), VARS2 (Mm00841195_m1), IARS2 (Mm01318288_m1), DARS2 (Mm00554081_m1), MARS2 (Mm00615287_s1), SARS2 (Mm00458332_m1), WARS2 (Mm04208966_m1), TARS2 (Mm00510878_m1) and HARS2 (Mm00475675_m1). The primer sequence for the iNOS mouse forward was GTTCTCAGCCCAACAATACAAGA and for iNOS mouse reverse was GTGGACGGGTCGATGTCAC. For eNOS mouse forward CTCCCAGCTGTGTCCAACAT. For eNOS mouse reverse CACACAGCCACATCCTCAAG. For NDUFB8 mouse forward TGTTGCCGGGGTCATATCCTA. For NDUFB8 mouse reverse AGCATCGGGTAGTCGCCATA. For MT-ND2 mouse forward TTGCGTGAGATTCGCGTTCA. For MT-ND2 mouse reverse ATTCGCGGATCAGAATGGGC. For SDHB mouse forward AATTTGCCATTTACCGATGGGA. For SDHB mouse reverse AGCATCCAACACCATAGGTCC. For UQCRC2 mouse forward AAAGTTGCCCCGAAGGTTAAA. For UQCRC2 mouse reverse GAGCATAGTTTTCCAGAGAAGCA. For MT-CO1 mouse forward ATTGGCAAGAGAGCCATTTCTAC. For MT-CO1 mouse reverse CACGCCGATCAGCGTAAGT. For ATP5A mouse forward TCTCCATGCCTCTAACACTCG. For ATP5A mouse reverse TCTCCATGCCTCTAACACTCG. For MT-ATP6 mouse forward AGGATTCCCAATCGTTGTAGCC. For MT-ATP6 mouse reverse CCTTTTGGTGTGTGGATTAGCA.

### 2.6 Mitochondrial isolation

Mitochondria were isolated from mouse skeletal muscle by homogenization using a polytron followed by a Douncer homogenizer with a Teflon pestle in homogenization buffer I (0.1 M KCl, 5 mM MgCl2, 5 mM EGTA, 5 mM sodium pyrophosphate, pH 6.8, and protease inhibitor tablet, Roche). Homogenates were centrifuged at 1,300×*g* for 10 min at 4 °C. The supernatant (SN1) was kept, and the pellet was resuspended in buffer II (0.25 M sucrose, 50 mM KCL, 5 mM EDTA, 1 mM sodium pyrophosphate, pH 7.4, and protease inhibitor tablet, Roche) and centrifuged at 1,300×*g* for 10 min at 4 °C. The supernatant (SN2), together with SN1 were centrifuged at 9,000×*g* for 15 min at 4 °C. The pellet (isolated mitochondria) was resuspended in buffer II.

### 2.7 Western blot

Homogenates for Western blot analyses were obtained from muscle. Tissues samples were homogenized in 10 vol of lysis buffer [10 mM Tris (pH 7.2), 150 mM NaCl, 1% Triton X-100, 0.1% SDS, 5 mM EDTA, 2 mM sodium orthovanadate, 50 mM NaF, 20 mM sodium pyrophosphate, and protease inhibitors mixture tablet (Roche)] with a mini-beadbeater (Biospec) twice for 30 s. Homogenates were rotated for 1 h at 4 °C in an orbital shaker and centrifuged at 16,000×*g* for 15 min at 4 °C. Supernatants were aliquoted and kept at −20 °C.

Proteins from total homogenates and the mitochondria isolation fraction were resolved in 10%, 12.5% or 15% acrylamide gels for SDS/PAGE and transferred to Immobilon membranes (Millipore). The following antibodies were used: Oxphos (abcam ab110413), ND2 (Proteintech 16879184), ATP6 (Millipore MABS1995), VDAC/Porin (Santa cruz 73614), Vinculin (Santa cruz 73614), LARS2 (Proteintech 17097-1-AP), TARS2 (Proteintech 15067-1-AP), NOS2 (Santa cruz sc-7271), NOS3 (BD Transduction 610296).

### 2.8 Statistical analysis

Microarray data was analysed as previously described [21], adjusting the data for batch and gender. Group comparisons in data other than microarray samples were performed using the Graphpad software. Human data were analysed as unpaired nonparametric distribution and the Mann-Whitney test was used. We assume a normal distribution of the data for animal studies using an unpaired parametric *t*-test. Data were presented as mean ± standard error unless otherwise stated. Significance was established at p < 0.05.

## 3 Results

### 3.1 Identification of the downregulated mitochondrial aminoacyl-tRNA synthetases gene expression in early-onset and classical forms of type 2 diabetes

**Fig. 1:**
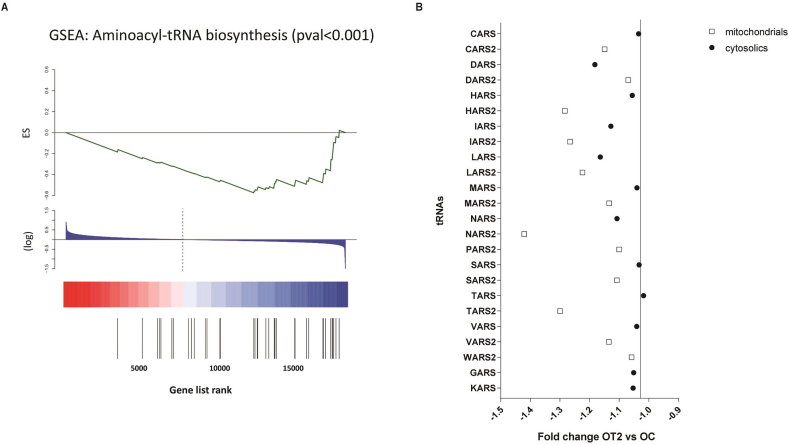
Identification of the downregulated aminoacyl-tRNA synthetases gene expression in early-onset and classical forms of type 2 diabetes. **(A)** The enrichment gene set expression of the aminocyl-tRNA synthetases. (**B)** Fold change OT2 vs OC of the main mitochondrial (identified with number 2) and cytosolic aminoacyl-tRNA synthetases.

Microarray analysis was accomplished to identify the global expression profile of muscles biopsies from control [young obese control (YC), and old obese control (OC)] and type 2 diabetic patients [early-onset type 2 diabetes (YT2), and classical T2D (OT2)] as previously reported [21]. The Gene Set Enrichment Analysis (GSEA) revealed the existence of genes dysregulated in both classical and early-onset type 2 diabetic patients that were enriched in metabolic pathways such as the metabolism of aminoacyl tRNA biosynthesis (Fig. 1A). As observed in Fig. 1B, the mitochondrial aminoacyl-tRNA synthetases (identified with number 2) were downregulated in type 2 diabetes; however, the cytosolic forms of the aminoacyl-tRNA synthetases were similar in type 2 diabetes and control subjects, showing that the defect in aminoacyl-tRNA synthetases is presumable mitochondrial.

### 3.2 Type 2 diabetes is characterized by reduced muscle expression of genes encoding mt-aaRSs

**Fig. 2:**
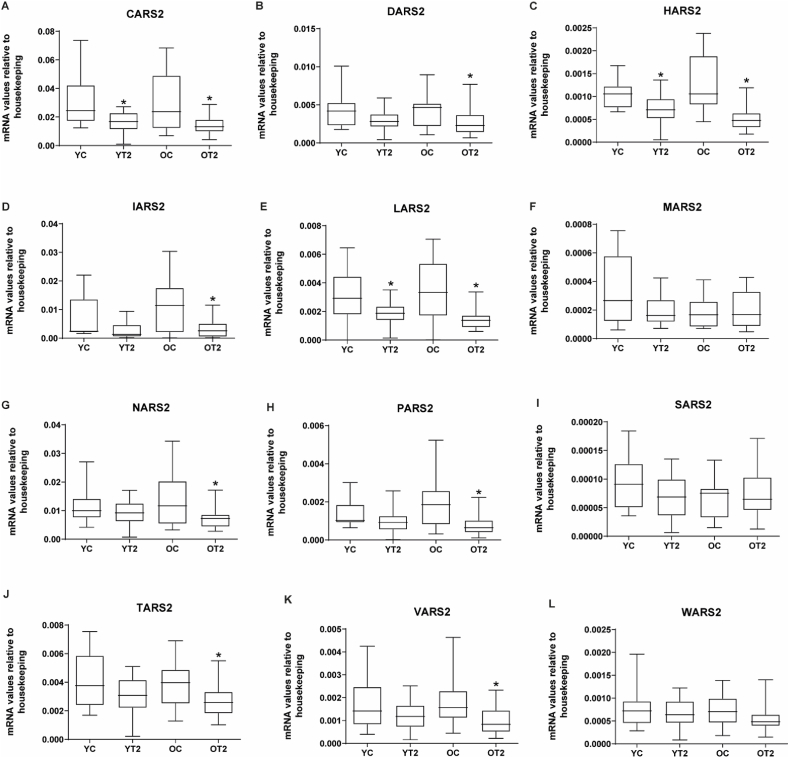
Skeletal muscle gene expression of mitochondrial tRNA synthetases in early-onset type 2 diabetic subjects (YT2) and late-onset type 2 diabetic subjects (OT2) and their respective matched control groups (YC and OC). Real-time PCR was performed in skeletal muscle biopsies. YT2 are early-onset type 2 diabetic subjects; OT2 are late-onset type 2 diabetic subjects, and their respective controls are YC and OC. **(A)** CARS2, **(B)** DARS2, **(C)** HARS2, **(D)** IARS2, **(E)** LARS2, **(F)** MARS2, **(G)** NARS2, **(H)** PARS2, **(I)** SARS2. Data are presented in boxplots; on each box, the central mark indicates the median, and the bottom and top edges of the box indicate the 25th and 74th percentiles, respectively. Unpaired non-parametric distribution and Mann-Whitney test was used *p < 0.05. YC (n = 12), YT2 (n = 21), OC (n = 17), OT2 (n = 24).

To validate the result obtained by microarrays, we next performed real-time PCR assays to analyze the expression of the mt-aaRSs in muscle from the control and type 2 diabetic groups. YT2 was characterized only by reduced expression of the mt-aaRSs CARS2, HARS2 and, LARS2 (Fig. 2A, C and, E). However, in type 2 diabetes of older subjects, reduced expression of CARS2, DARS2, HARS2, IARS2, LARS2, NARS2, PARS2, TARS2, and VARS2 was detected (Fig. 2. A-E, G-H and J-K). No change in any of the conditions was observed in the case of MARS2, SARS2, and WARS2 (Fig. 2. F, I and L).

**Fig. 3:**
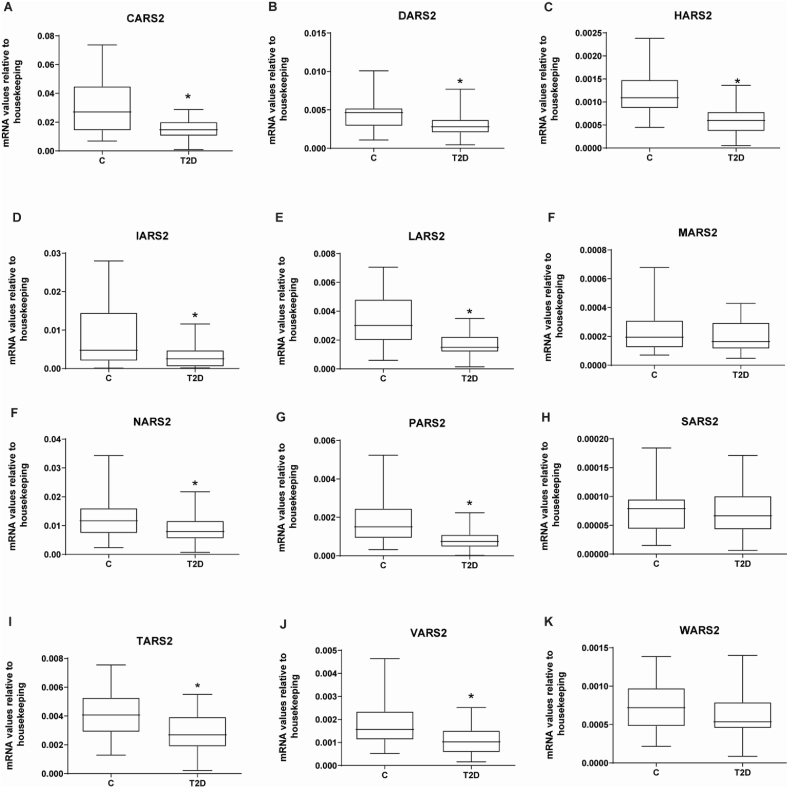
Skeletal muscle gene expression of mitochondrial tRNA synthetases in type 2 diabetic subjects (T2D) and their respective matched control groups. Real-time PCR was performed in skeletal muscle biopsies. T2D are type 2 diabetic subjects and their respective matched control groups. **(A)** CARS2, **(B)** DARS2, **(C)** HARS2, **(D)** IARS2, **(E)** LARS2, **(F)** MARS2, **(G)** NARS2, **(H)** PARS2, **(I)** SARS2. Data are presented in boxplots; on each box, the central mark indicates the median, and the bottom and top edges of the box indicate the 25th and 74th percentiles, respectively. Statistical analyses comparing type 2 diabetic subjects vs respective controls were performed by unpaired *t*-test *p < 0.05. C (n = 29), T2D (n = 45).

To be sure that the changes in type 2 diabetes excluded changes in mt-aaRSs associated with methionine, serine and tryptophan, we decided to unify the data by obese phenotype and T2D (independently of age). In this context, general type 2 diabetes (YT2+ OT2) was characterized by reduced expression of genes encoding for the mt-aaRSs: CARS2, DARS2, HARS2, IARS2, LARS2, NARS2, PARS2, TARS2, and VARS2 (Fig. 3. A-E, G-H and J-K). However, no changes were observed in the mRNA expression of MARS2, SARS2, or WARS2 in the diabetic groups (Fig. 3. F, I, and L) as observed in separate groups of diabetic patients.

### 3.3 Mt-aaRSs are dysregulated in diabetic obese mice

To further explore the link between mt-aaRSs expression and T2D, we employed a series of mouse models. Given that all human subjects (both diabetic and non-diabetic) in this study are obese, we wanted to i) verify that diabetic obese mice recapitulate the mt-aaRSs expression patterns observed in obese T2D patients, and ii) evaluate the contribution of obesity towards mt-aaRSs expression in diabetic and non-diabetic contexts.

**Fig. 4:**
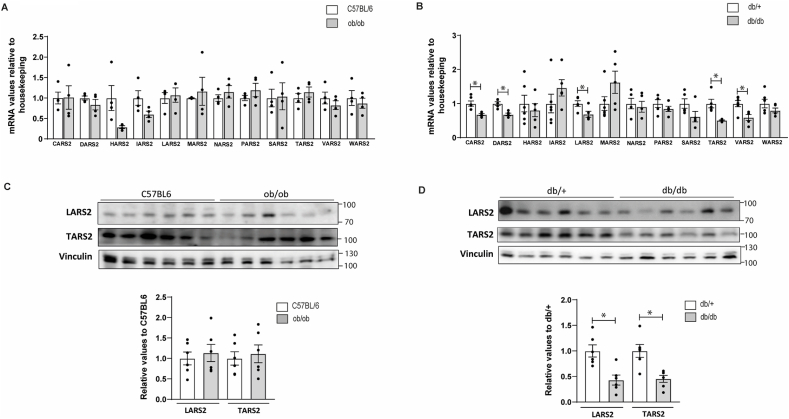
Skeletal muscle gene and protein expression of mitochondrial tRNA synthetases in obese and diabetic mice and their respective matched control group. Real-time PCR and Western blot were performed in skeletal muscle biopsies. Ob/ob are obese mice, db/db are diabetic mice, and their respective controls are C57BL/6 and db/+. **(A)** Skeletal muscle gene expression of mt-aaRSs in obese mice. **(B)** Skeletal muscle gene expression of mt-aaRSs in diabetic mice. **(C)** Skeletal muscle protein expression of LARS2 and TARS2 in obese mice. **(D)** Skeletal muscle protein expression of LARS2 and TARS2 in diabetic mice. Data are mean ± SEM. Statistical analyses comparing C57BL6 vs ob/ob or db/+ vs db/db mice were performed by unpaired *t*-test *p < 0.05. n = 4–6/group.

We employed the well-established mouse models ob/ob (obese, insulin-resistant, metabolically non-diabetic) and db/db (obese, insulin-resistant, metabolically diabetic) [24]. Note that these two mouse models have different genetic backgrounds (ob/ob: C57BL/6 carrying a mutation in the leptin gene; db/db: C57BLKS/J carrying a mutation in the leptin receptor gene). Therefore, we performed comparative analyses of mt-aaRSs expression in ob/ob mice relative to non-diabetic lean wild-type mice (C57BL/6); and an equivalent analysis in db/db mice against their diabetic lean siblings (db/+). We did not observe a statistically significant differential expression of mt-aaRSs in ob/ob mice compared to C57BL/6 mice (Fig. 4A), indicating that downregulation of mt-aaRSs is not associated with obesity in a non-diabetic context. However, we found a statistically significant downregulation of CARS2, DARS2, LARS2, TARS2, and VARS2 in db/db mice with respect to db/+ mice (Fig. 4B). Together, this data showed that downregulation of mt-aaRSs in diabetic obese individuals is a conserved signature in mice and human patients.

### 3.4 Mitochondrial aminoacyl-tRNA synthetases expression is impaired in diabetic mice and leads to an imbalanced in mitochondrial OXPHOS complex formation

Reduced expression of mt-aaRSs should impact mitochondrial translation. We analysed the amino acid composition of the 13 well-validated proteins encoded and translated in animal mitochondria [25]. These proteins are highly conserved in humans (Hs) and mice (Mm). We found them to be primarily enriched in amino acids Leu (17% Hs/15.5% Mm), Ile (8.42% Hs/9.79% Mm), and Thr (9.26% Hs/8.12% Mm) (Suppl. Table 2). Notably, these amino acids are charged into mt-tRNAs by three of the most downregulated mt-aaRS that we detected in obese T2D patients (i.e. LARS2, IARS2, and TARS2, respectively) (Fig. 3E, D, J). In the case of the diabetic mice (db/db) only the gene expression of LARS2 and TARS2 was downregulated (Fig. 4B). Moreover, the protein expression of LARS2 and TARS2 was decreased in the skeletal muscle of diabetic mice (Fig. 4D) while no differences were found in ob/ob mice (Fig. 4C).

Mitochondrial-encoded proteins are involved in the formation of the mitochondrial electron transport chain (OXPHOS) complex [26]. Therefore, aberrant mitochondrial protein synthesis due to defects in mitochondrial aminoacyl-tRNAs could impact OXPHOS complex formation. To assess the effects of lower expression of mt-aaRSs in the skeletal muscle of db/db mice on the OXPHOS biogenesis, we carried out western blotting analysis to examine the levels in 7 subunits of OXPHOS complexes in the skeletal muscle of db/db, ob/ob, and their respective control mice. These subunits included three mtDNA-encoding polypeptides (NADH:Ubiquinone Oxidoreductase Core Subunit 2 (ND2), cytochrome *c* oxidase subunit 1 (CO1) and ATP synthase subunit 6 (ATP6)), and four nucleus-encoding proteins (NADH:Ubiquinone Oxidoreductase Subunit B8 (NDUFB8), succinate dehydrogenase iron-sulfur subunit (SDHB), Ubiquinol-Cytochrome C Reductase Core Protein 2 (UQCRC2) and ATP synthase F1 subunit alpha (ATP5A)).

**Fig. 5:**
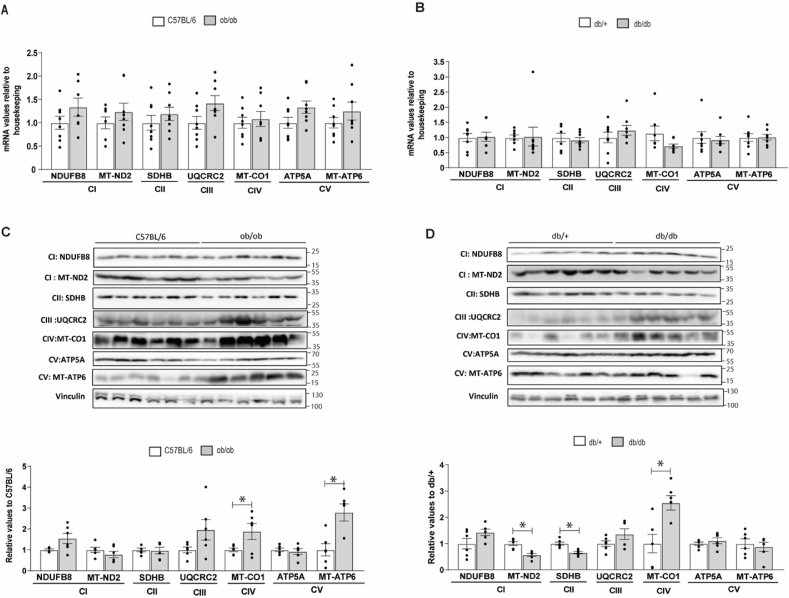
Skeletal muscle gene and protein expression of mitochondrial complexes in obese and diabetic mice and their respective matched control group. Real-time PCCR and Western blot were performed in skeletal muscle biopsies. Ob/ob are obese mice, db/db are diabetic mice, and their respective controls are C57BL/6 and db/+. **(A)** Skeletal muscle gene expression of mitochondrial complexes in obese mice. **(B)** Skeletal muscle gene expression of mitochondrial complexes in diabetic mice. **(C)** Skeletal muscle protein expression of mitochondrial complexes in obese mice. **(D)** Skeletal muscle protein expression of mitochondrial complexes in diabetic mice. Data are mean ± SEM. Statistical analyses comparing C57BL6 vs ob/ob or db/+ vs db/db mice were performed by unpaired *t*-test *p < 0.05. n = 6–8/group.

In obese (ob/ob) and diabetic (db/db) mice, the skeletal muscle gene expression of NDUFB8, MT-ND2, SDHB, UQCRC2, MT-CO1, ATP5 and, MT-ATP6 was similar to the corresponding control mice (Fig. 5A and B). Moreover, in obese mice, an upregulation of the protein expression of mitochondrial subunits MT-CO1 (Complex IV) and MT-ATP6 (Complex V) was observed (Fig. 5C). No changes in the protein expression of the subunits NDUFB8 and mitochondrial MT-ND2 (Complex I), SDHB (Complex II), UQCRC2 (Complex III) and ATP5A (Complex V) were detected (Fig. 5C). By contrast, in db/db mice, alterations in the protein expression of three subunits of the OXPHOS complexes were detected (Fig. 5D). These included downregulation of the subunits mitochondrial MT-ND2 (Complex I) and SDHB (Complex II); and upregulation of mitochondrial MT-CO1 (Complex IV) (Fig. 5D). Note that SDHB is a nuclear-encoded mitochondrial protein and therefore, their protein levels should not be directly dependent on mt-aaRS expression. However, MT-ND2 and MT-CO1 are encoded and translated in the mitochondria. We found a ∼55% reduction in MT-ND2 protein levels in db/db mice compared to db/+ mice, consistent with impaired mitochondrial translation in obese diabetic mice. No changes were observed in the subunits NDUFB8 (Complex I), UQCRC2 (Complex III), ATP5A, and MT-ATP6 (Complex V) between db/db and db/+ mice. However, obesity caused a considerable increase in MT-ATP6 that is no longer observed in db/db mice.

### 3.5 iNOS abundance is increased in mitochondrial fractions obtained from skeletal muscle in diabetic mice

**Fig. 6:**
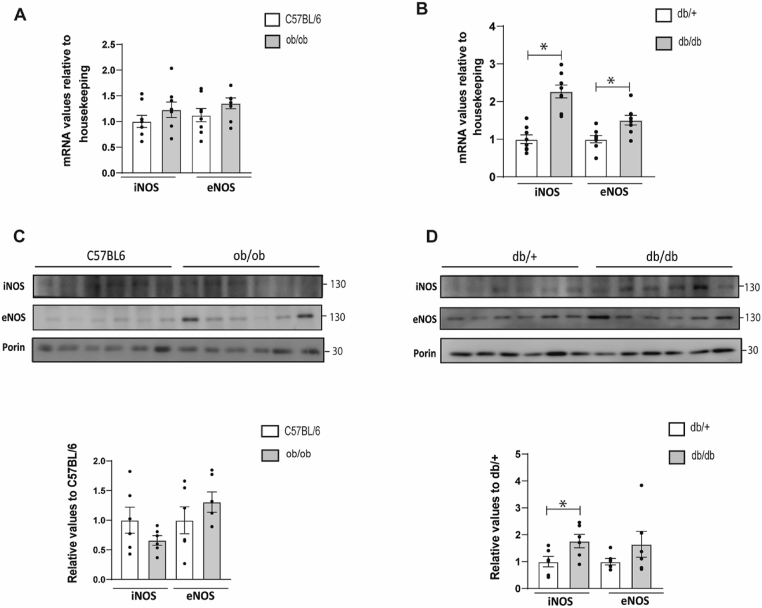
Skeletal muscle gene expression of iNOS and eNOS in obese and diabetic mice and their respective matched control group. iNOS and eNOS protein expression in mitochondria isolated from skeletal muscle of obese and diabetic mice and their respective matched control group. Real-time PCR or Western blot was performed in skeletal muscle biopsies. Ob/ob are obese mice, db/db are diabetic mice, and their respective controls are C57BL/6 and db/+. **(A)** Skeletal muscle gene expression of iNOS and eNOS in obese mice. **(B)** Skeletal muscle gene expression of iNOS and eNOS in diabetic mice. **(C)** iNOS and eNOS protein expression in enriched mitochondrial fractions from skeletal muscle of obese mice. **(D)** iNOS and eNOS protein expression in enriched mitochondrial fractions from skeletal muscle of diabetic mice. Data are mean ± SEM. Statistical analyses comparing C57BL6 vs ob/ob or db/+ vs db/db mice were performed by unpaired *t*-test *p < 0.05. n = 6–8/group.

It has been reported that nitrosative stress inhibits aminoacylation of mitochondrial threonyl-tRNA and leucyl-tRNA synthetases (TARS2 and LARS2) by S-nitrosation [18]. Nitric oxide is synthesized enzymatically from l-arginine through the actions of the nitric oxide synthases (NOS). Therefore we measured if NOS expression was dysregulated in diabetes or obesity. iNOS and eNOS gene expression was higher in the skeletal muscle of diabetic mice (Fig. 6B), and not in obese mice (Fig. 6A). Moreover, iNOS protein was enhanced in mitochondrial-enriched fractions obtained from diabetic mice (Fig. 6D), whereas no differences were observed in obese mice (Fig. 6C).

Altogether our data suggest that an impaired translation of mitochondrial-encoded proteins leading to an imbalance in the formation of mitochondrial OXPHOS complexes and mitochondrial dysfunction, which is one of the most important defects found in skeletal muscle from type 2 diabetic patients.

## 4 Discussion

In this study, we focused our attention on mt-aaRSs, which constitute a class of enzymes that control cellular protein homeostasis in the mitochondria. Our results establish a diabetic signature: reduced expression of mt-aaRSs in type 2 diabetic skeletal muscle that is applicable independently of the age of diabetes onset and in a diabetic mice model.

Mt-aaRSs participate in the first step of protein biosynthesis charging mitochondrial tRNAs with their cognate amino acids. Mitochondrial protein biosynthesis is a critical process because the human mitochondrial genome encodes 13 proteins, which are all essential members of the OXPHOS complex. A downregulation of mt-aaRSs could be affecting the synthesis of the mitochondrial subunits of OXPHOS complexes in the skeletal muscle of diabetic patients. Recently, data from the largest proteomic analysis of human skeletal muscle reveal the downregulation of proteins of OXPHOS complexes in prediabetes and T2D [9]. Among these proteins that were downregulated in T2D samples, seven were mitochondrial genome encode proteins (MT-ATP6, MT-CO1, MT-CO2, MT-ND2, MT-ND4, MT-ND5 and MT-CYB), and 49 were nuclear genome encode proteins, suggesting that reduced expression of mt-aaRSs could impact mitochondrial translation in the skeletal muscle of T2D patients.

Strikingly, in our results, a nuclear genome-encoded protein SDHB (Complex II) was downregulated in the skeletal muscle of diabetic mice. These results agree with a DARS2 (mitochondrial aspartyl-tRNA synthetase) knockout mice specifically in the skeletal muscle [27]. These mice showed a substantial decrease in COX1 and COX4-1 (Complex IV), both of them mitochondrial-encoded proteins, and moderate reductions in the levels of NDUFA9 (Complex 1) and UQCRC2 (Complex 3), both of them nuclear-encoded proteins in the skeletal muscle. However, they showed a strong upregulation of MT-ND2 a mitochondrially encoded subunit that completely lacks aspartate residues [27]. Also, YARS2 (mitochondrial tyrosyl-tRNA synthetase) knockout (KO) HeLa cells presented aberrant tRNA^Tyr^ aminoacylation and reductions in the levels of mitochondrion- and nucleus-encoding subunits of OXPHOS. Furthermore, YARS2 ablation caused defects in the stability and activities of OXPHOS complexes [28].

Our results also showed that in diabetic mice, a downregulation of mt-aaRSs was affecting the synthesis of the mitochondrially encoded MT-ND2 (Complex I). This subunit in mice is primarily enriched in the amino acid leucine (18,44%) and threonine (12,39%) (Suppl. Table 3). Then, the reduction of MT-ND2 correlates with the decrease in LARS2 and TARS2 protein expression that was also found in the muscle of diabetic mice. In addition, the MT-ATP6 subunit also has an important enrichment of leucine (19,47%) and threonine (11,50%) (Suppl. Table 3) and in diabetic mice did not show that considerable increased observed in ob/ob mice. This implies that MT-ATP6 is also affected by the decreased protein expression of LARS2 and TARS2 observed in db/db mice. By contrast, the contribution of threonine (6,6%) (Suppl. Table 3) is lower in the subunit MT-CO1. This observation suggests an attempt to compensate for OXPHOS defects associated with impaired mitochondrial translation by stimulating mitochondrial biogenesis as previously reported in tissues of mitochondrial disease patients [29]. However, MT-CO1 is the main subunit of the cytochrome *c* oxidase complex and is the last enzyme in the mitochondrial electron transport chain, which drives oxidative phosphorylation where O_2_ is converted to H_2_O. In obesity condition, the upregulation of both MT-CO1 and MT-ATP6 subunits suggets more activity of the respiratory chain. More oxygen converted to H_2_O and more ATP production. However in diabetic condition, there is a lack of protons due to defects in Complex I and II, the overactivation of Complex IV and non enough activity of Complex V to generate ATP, will induce a release of oxygen that can be used to produce reactive oxygen species (ROS). An enhanced level of ROS could induce an overproduction of NO, which in turn, reacting with ROS, produces reactive peroxynitrite (ONOO^−^), causing mitochondrial DNA breakage and mitochondrial damage [30] as a vicious cycle.

Nitric oxide (NO) is a key signalling molecule in several cellular pathways and plays an important role in physiology and pathophysiology [31]. There is evidence that mitochondria produce NO [32] and NOS proteins have been detected in mitochondria, although their identity is debated [33,34]. We have detected iNOS and eNOS protein expression in mitochondrial-enriched fractions of from mouse skeletal muscle. These results agree with several studies that demonstrate mitochondrial co-localization of the immunoreactivity using antibodies against iNOS [35] and eNOS [36]. eNOS is constitutive and is activated by interactions with calcium and calmodulin, while iNOS is calcium-independent and is usually activated in defense responses such as infection and inflammation [37]. eNOS produces lower concentrations of NO for short periods, while iNOS can produce high amounts of NO during long periods [31]. NO can promote nitrosative modifications, which will act in physiological responses through signaling or promoting mitochondrial damage [31]. It has been reported that nitrosative stress inhibits the aminoacylation of mitochondrial threonyl-tRNA and leucyl-tRNA synthetases (TARS2 and LARS2) by S-nitrosation [18]. In this regard, we have detected higher iNOS levels in mitochondrial-enriched fractions from diabetic mice, suggesting an enhanced NO production, and subsequent nitrosative stress. However, we cannot rule out the possibility that ER- mitochondrial associated membranes (MAMs) attached to mitochondria are the source of NO, thus influencing by proximity mitochondrial biology. Finally, we also hypothesize that the NO produced at the mitochondria or on its proximity are involved in the downregulation of TARS2 and LARS2 protein levels and with the decreased expression of mt-aaRSs due to NO action in DNA breakage. However, further studies are necessary to proof a role of iNOS by the use of specific inhibitors, in order to validate this statement.

## Funding

The project was funded with a grant to JJN at Trinity College Dublin from 10.13039/501100001648EFSD/Novo Nordisk Clinical Research Program in Adolescents with Type 2 Diabetes (Cellular Mechanisms of Insulin Resistance in Early Onset Type 2 Diabetes, grant reference T04001), Grant 2017SGR1015 from the “Generalitat de Catalunya”, 10.13039/501100013941CIBERDEM (“Instituto de Salud Carlos III”), 10.13039/501100004837MCIN (PID2019-105466RA-I00 MCIN/AEI/10.13039/501100011033, RYC2018-024345-I MCIN/AEI/10.13039/501100011033 y por El FSE invierte en tu futuro, and PID2019-106209RB-I00 MCIN/AEI/10.13039/501100011033), “la Caixa” Foundation, 10.13039/100005622Health Research Grant 2021 (LCF/PR/HR21/52410007), 10.13039/501100005142Fundación BBVA, 10.13039/501100001648EFSD, and AFM Téléthon. 10.13039/100007678IRB Barcelona is the recipient of a Severo Ochoa Award of Excellence from MINECO (Government of Spain). A.Z. is a recipient of an 10.13039/501100003741ICREA “Academia” Award (Generalitat de Catalunya).

## Contribution statement

IL-S, AD-R, and MIHA conceived the study. IL-S, AD-R, RV, IM-R, AD-R, EP, DC, AP, KW, DOH, DO, TC, LR, JJN and MIH-A performed the experiments. IL-S, AD-R, RV, IM-R, EP, TC, LR, A-Z and MIH-A analysed the data. IL-S and MIH-A wrote the manuscript and all authors contributed to editing. A-Z and MIH-A are the guarantors of this work.

## Declaration of competing interest

The authors are not affected by any conflict of interest.

## Data Availability

Data will be made available on request.
