# Supplementary material for: Decreased expression of mitochondrial aminoacyl-tRNA synthetases causes downregulation of OXPHOS subunits in type 2 diabetic muscle
Source: Redox Biol. 2023 Feb 8;61:102630. doi: 10.1016/j.redox.2023.102630 (PMC9958393; doi:10.1016/j.redox.2023.102630)
Supplement: Multimedia component 2 [file mmc2.docx]

**Supplementary Table 2. Total Amino Acid composition of the 13 well-validated proteins that are encoded and translated in human and mouse mitochondria.**

**Supplementary Table 3. Amino acid composition of each of the 13 proteins encoded in mouse mitochondria**
